# Two newly recorded genera and species of Owlflies (Neuroptera: Ascalaphidae) from China

**DOI:** 10.3897/BDJ.4.e7451

**Published:** 2016-01-18

**Authors:** Ming xue Yang, Xin li Wang, Ming xia Sun

**Affiliations:** ‡ China Agricultural University, Beijing, China; § Department of Entomology, China Agricultural University, Beijing, China; | Institute of Zoology, Chinese Academy of Science, Beijing, China

**Keywords:** *
Bubopsis
*, *
Nousera
*, Ascalaphidae, China

## Abstract

**Background:**

The records of genus *Bubopsis* McLachlan, 1898 with species *Bubopsis tancrei* Weele, 1908 and the genus *Nousera* Navás, 1923 with species *Nousera gibba* Navás, 1923 have not been published in China.

**New information:**

The genus *Bubopsis* McLachlan, 1898 with species *Bubopsis tancrei* Weele, 1908 and the genus *Nousera* Navás, 1923 with species *Nousera gibba* Navás, 1923 are recorded for the first time from China. We provide detailed descriptions and illlustrations of specimens and the collecting information of the specimens are also provided.

## Introduction

Sziráki (1998) proposed the monotypic genus *Nousera* Navás, 1923 is a junior synonym of *Pseudoptynx* Van der Weele, 1908. In contrast, Ábrahám and Mészáros (2006) thought that *Nousera* should be a valid genus name. In the revision on the genus of *Nousera*, Ábrahám compared the morphological differences between *Nousera* and *Pseudoptynx* and described a new species *Nousera herczigi* from Pakistan. Meanwhile he gave *Pseudoptynx* a new name *Ascapseudoptynx*, because *Pseudoptynx* Van der Weele, 1908 is a junior homonymy of *Pseudoptynx* Kaup, 1848 in Aves (Oswald and Penny 1991). So the genus *Nousera* has only two species now, mainly distributed in Laos, Thailand, Pakistan, Malaysia (Ábrahám and Mészáros 2006, Michel 2005). Until now, this genus has not been recorded from China.

The genus *Bubopsis* McLachlan, 1898 comprises seven species, mainly distributed in Southern Europe, North Africa, Middle East, Southern Arabia Peninsula, Central Asia and India (Alexandrov-Martynov 1926, Aspöck et al. 1980a, Aspöck et al. 1980b, Aspöck et al. 1978, Hölzel 2004, McLachlan 1898, Sziráki 1998, Sziráki 2000, van der Weele 1908). Prior to our study *B. tancrei* Weele, 1908 was known only from Central Asia (Sziráki 2000).

## Materials and methods

Terminology of wing venation follows Wang (2003), while genitalia terminology follows Tjeder (1977).

Photographs of morphological characteristics were taken with a Canon EOS 500D digital camera connected to an Olympus U-CTR30-2 microscope, using UV-C (Application Suite) applied software by United Vision Ltd. Photographs of habitus were taken by a Canon EOS 60D digital camera. All figures were processed in Adobe Photoshop CS5.

All specimens examined are deposited in the Insect Collections of China Agricultural University (ICCAU), Beijing, China.

**Abbreviations:** dv = distivalvae; ep = ectoprocts; gc = gonarcus-paramere complex; lg = linguella; pa = parameres；pv = pulvini; s = sternite; t = tergite; vv = ventrovalvae.

## Taxon treatments

### Nousera

Navás, 1923

Nousera Navás, 1923: 5. Navás 1923
Nousera Nousera gibba Navás, 1923

#### Diagnosis

Eyes divided by a transverse furrow almost equally. Antenna not reaching to pterostigma of forewing. The wings narrow, axillary angle of forewing with triangular projection, apical area beyond the vein Sc+R with three rows of cells. Hindwing shorter than forewing, axillary angle with lobe-like projection, apical area beyond the vein Sc+R with two or three rows of cells, CuA area narrow with 1 or 2 rows of cells, CuA2 short and indistinct, CuP reaching to the posterior margin of hindwing, not intersecting with CuA2. Male abdomen longer than hindwing, female abdomen shorter than hindwing. The anterior part of 2^nd^ tergite of male abdomen elevated. Ectoprocts of male and female not extended.

### Nousera gibba

Navás, 1923

Nousera gibba Navás, 1923: 5.

#### Diagnosis

Wings elongated and narrow, hyaline, axillary angle of forewing with triangular projection, CuA area of hindwing with one row of cells. The anterior part of 2^nd^ tergite of male abdomen elevated.

##### Description

**Male** Fig. 1

**Figure 3.**
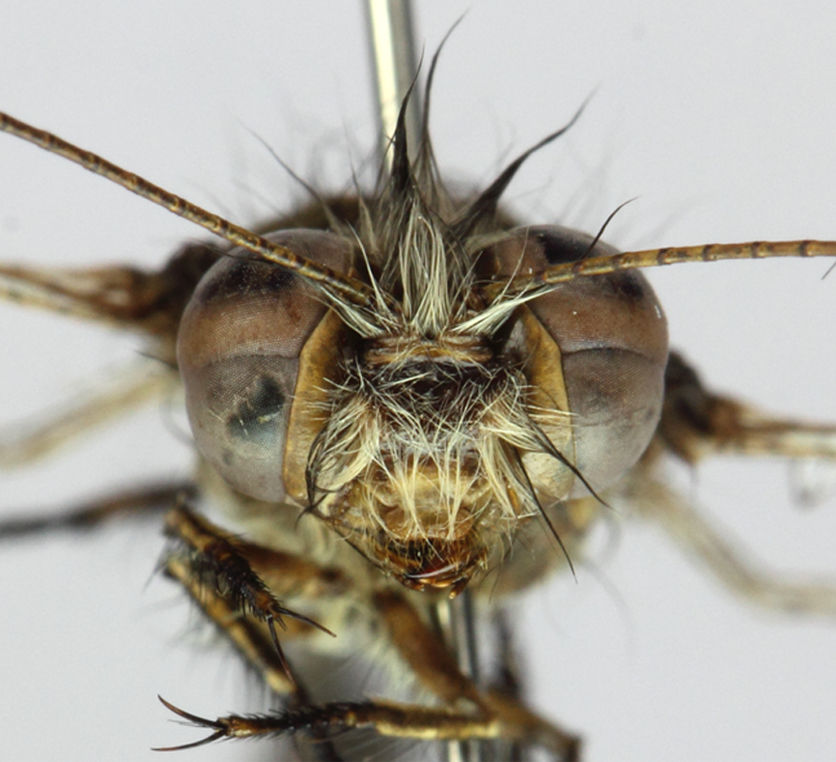
*Nousera gibba* Navás, 1923, Head of male, front view.

**Head** Fig. 3 Vertex reddish brown with long soft pale yellow and brown hairs. Frons dark-brown with long pale yellow hairs. Gena yellowish-brown, hairless. Clypeus yellowish-brown with sparse pale yellow hairs on the lateral margins. Labrum yellowish-brown with sparse yellow hairs on the ventral margin. Mandible reddish brown, but black distally. Maxillae and labial palpi reddish brown with rather short brown setae. Occiput yellowish-brown. Eyes divided by a transverse furrow almost equally. Antennae as long as 2/3 of the forewing, brown but with the basal 1/3 yellowish-brown; club long pyriform, brown with narrow black rings, and a yellowish-brown spot ventrally, the setae on club rather short and black.

**Thorax.** Pronotum narrow, yellowish-brown centrally and brown laterally. Mesonotum yellowish-brown, prescutum and scutellum with two black spots, mesoscutum dark-brown with two small yellowish-brown spots. The hairs on whole mesonotum sparse yellowish-brown. Metanotum dark-brown with long soft dark-brown hairs. The lateral and ventral parts of thorax yellowish-brown with pale hairs.

**Wings.** Membrane hyaline and with brown veins. Pterostigma black-brown with 4 cross-veins. Forewing: Basal part narrow, axillary angle with triangular projection; apical area beyond the vein Sc+R with 3 rows of cells; CuA area with 4 rows of cells. Hindwing: Basal part narrow, anal area with lobe-like projection and the edge of projection with long soft hairs; apical area beyond the vein Sc+R with 3 rows of cells, but only 1-3 cells in the middle row; CuA area rather narrow with only 1 row of cells.

**Legs.** Coxa and trochanter reddish brown with soft white hairs. Femur reddish brown, dark-brown distally with long pale yellow hairs. Tibia dark-brown with reddish brown stripe longitudinally outside, setae on it long and black ， two spurs reddish brown, as long as claws. Tarsal segments 1-4 dark-brown, segment 5 dark-brown, but reddish brown distally, tarsal setae dark-brown. Claws rather long, black, reddish brown distally.

**Figure 4.**
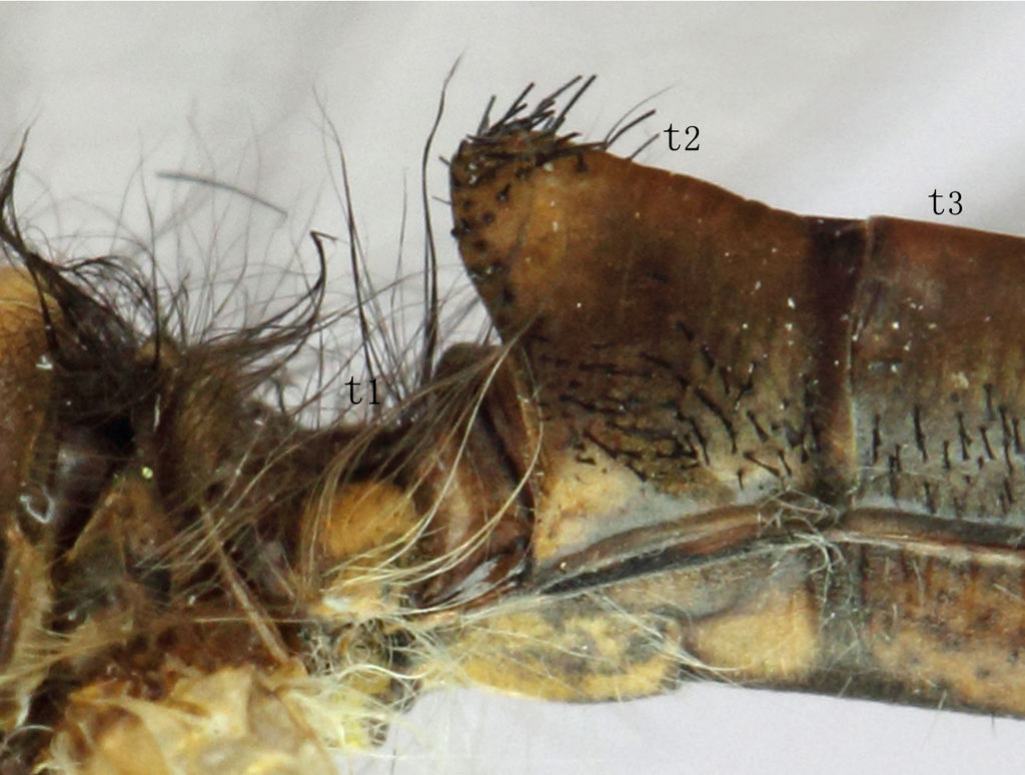
*Nousera gibba* Navás, 1923, Basal part of abdomen of male, lateral view.

**Abdomen.** Longer than hindwings. The first tergite hollowed, brown with two long yellowish-brown spots on both sides, hairs on it long soft. The anterior part of 2^nd^ tergite elevated Fig. 4, brown but the anterior margin reddish brown with black setae. 3-8 tergites ochre-brown with short black setae laterally. Sternites 1-4 yellowish-brown each with a dark-brown, cross-shaped mark. Sternites 6-8 pale yellow with short white hairs.

**Male genitalia.** Ectoprocts brown, covered with long black setae Fig. 6. Sternite 9 covered with long black setae on posterior margin. Pulvini pale brown with several long yellow gonosetae. Gonarcus pale brown, distally bearing one row of teeth on each lateral margin Fig. 7. Parameres less developed. Pelta absent. **​**

**Female** Fig. 2

**Size.** Body length 25 mm. Antennae length 22-24 mm. Forewing length 31-32 mm, width 6-7 mm. Hindwing length 28 mm, width 5 mm. Abdomen length 18 mm.

**Figure 5.**
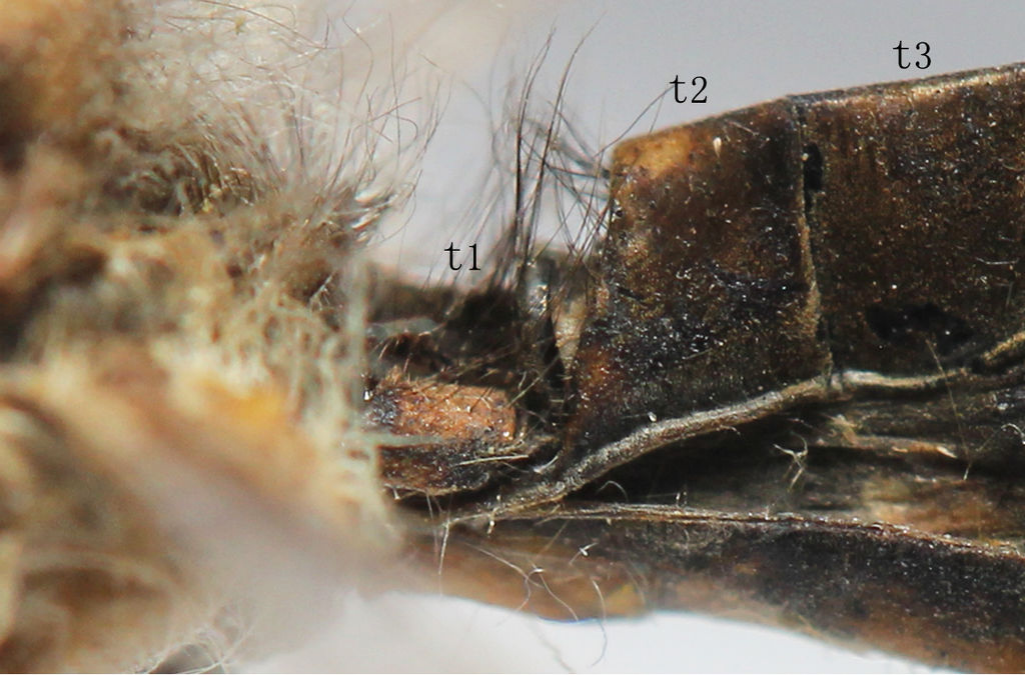
*Nousera gibba* Navás, 1923, Basal part of abdomen of female, lateral view.

**Figure 6.**
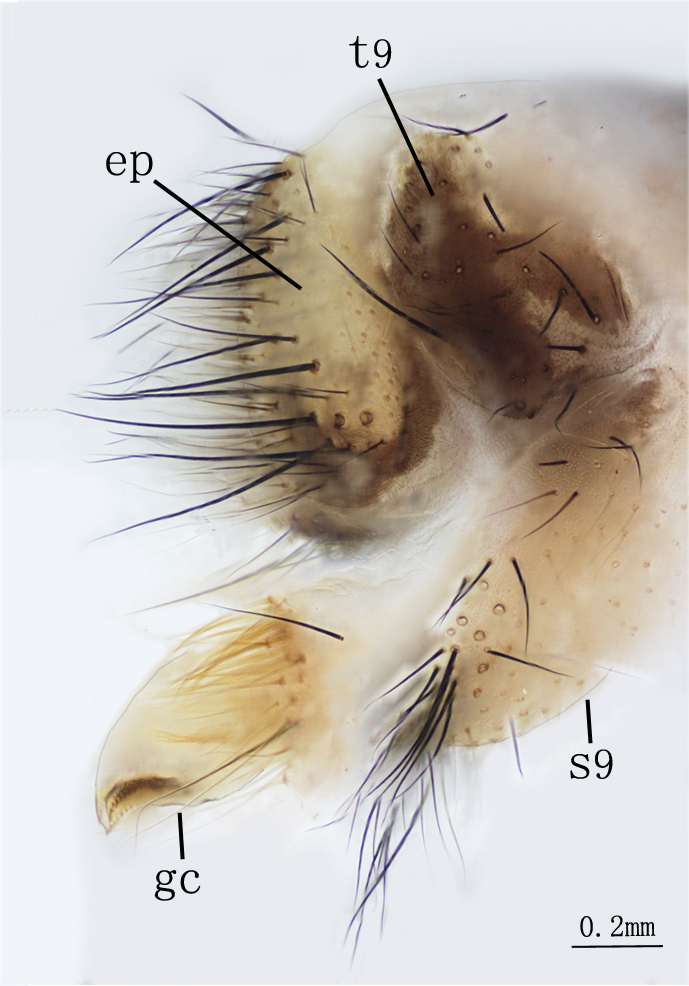
*Nousera gibba* Navás, 1923, Abdomen terminal of male with gonarcus-paramere complex, lateral view.

**Figure 7.**
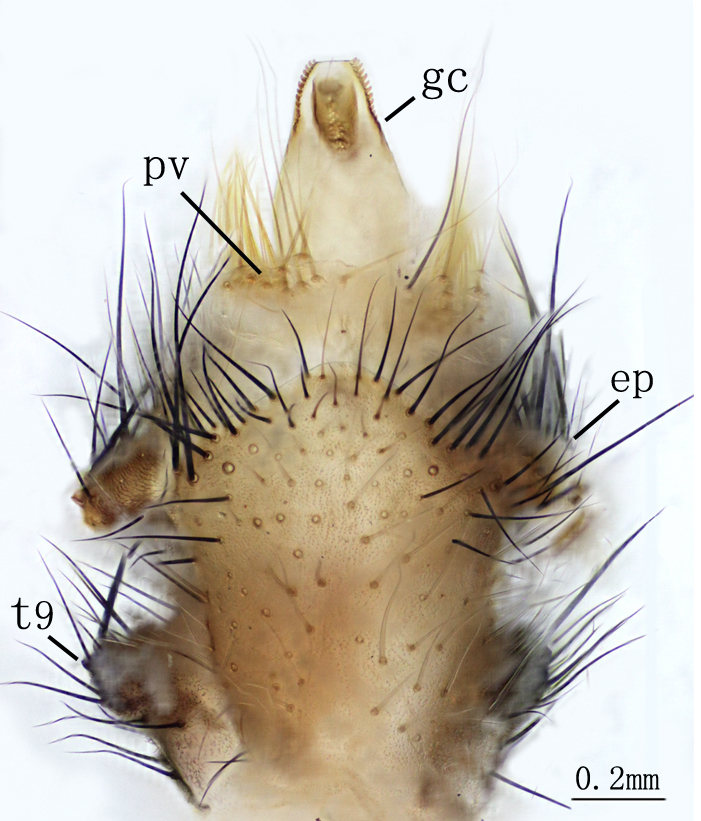
*Nousera gibba* Navás, 1923, Abdomen terminal of male with gonarcus-paramere complex, ventral view.

The Abdomen is shorter than hindwings. The 1^st^ tergite lower slightly, the 2^nd^ tergite not elevated Fig. 5.

**Figure 8.**
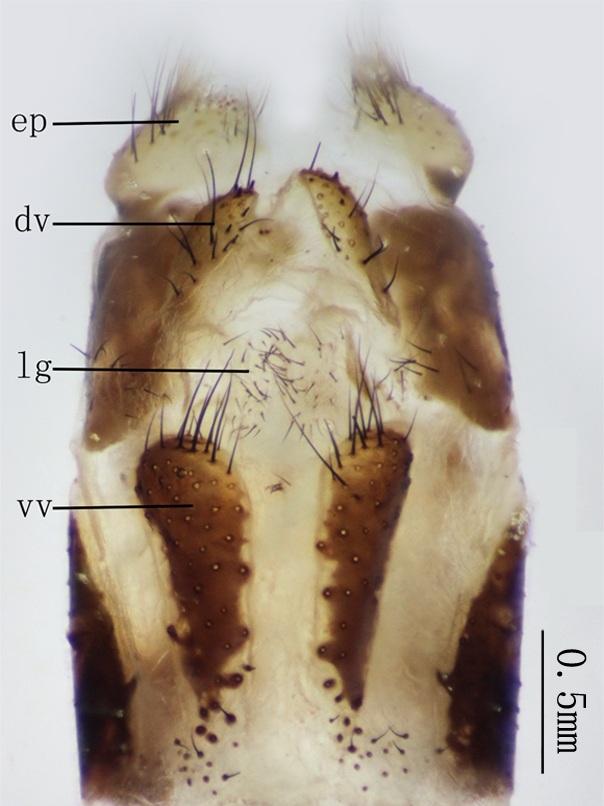
*Nousera gibba* Navás, 1923, Abdomen terminal of female, ventral view.

**Figure 9.**
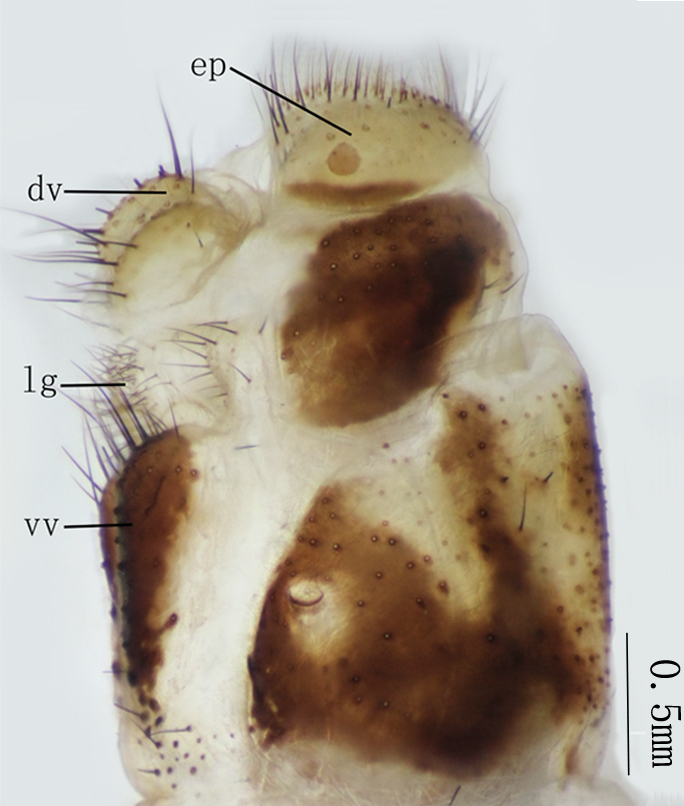
*Nousera gibba* Navás, 1923, Abdomen terminal of female, lateral view.

**Female genitalia** Figs 8, 9. Ectoprocts sub-elliptical in lateral view, yellow with brown setae. Distivalvae semi-circular, brown with long brown setae. Linguella slightly chitinized, transparent with short brown hairs. Ventrovalvae dark brown with long setae. Interdens absent.

#### Distribution

China: Yunnan; Laos, Malaysia, Thailand, Vietnam.

#### Material examined

1♀, Mengla Yaoqu, Yunnan Prov. (light trap), 6-V-2005, Liu Xingyue (CAU-N200555); 1♀, Shilin County Heilongtan, Yunnan Prov., 2-Ⅴ-2013; 1♂, Yongde County Wumulongxiang, Yunnan Prov., 22-Ⅳ-22; 4♂, Shilin County Heilongtan, Yunnan Prov., 2-Ⅴ-2013; 1♂, Tengchong Zhengding, Yunnan Prov., 3-Ⅴ-2013.

### Bubopsis

McLachlan, 1898

Bubo Rambur, 1842: 353. Rambur 1842
Bubopsis McLachlan, 1898: 159.
Bubopsis Bubopsis agrionoides (Rambur, 1838)

#### Diagnosis

Eyes divided by a transverse furrow almost equally. Antennae almost as long as 2/3 of forewing. Wings elongated and narrow with rounded apex, the anterior margin and outer margin of forewing almost parallel, axillary angle obtuse, slightly prominent. Hindwing shorter than forewing. Abdomen in both sexes shorter than hindwing. Ectoprocts of male about as long as 6-9 abdominal segments together with a short, rod-shaped branch downward.

### Bubopsis tancrei

Weele, 1908

Bubopsis tancrei Weele, 1908: 273.

#### Diagnosis

Wings hyaline, veins of C, M, CuP yellow. Abdomen yellow with black markings. Ectoprocts of male elongate slightly bent with a rod-shaped branch downward.

##### Description

**Male** Fig. 10

**Size.** Body length 28-29 mm. Antennae lack. Forewing length 28 mm. Hindwing length 25 mm. Abdomen length 17 mm.

**Head.** Vertex brown with long dense soft brown and white hairs. Frons brown and gena yellow both with long dense soft pale yellow hairs. Clypeus and labrum yellow with short sparse pale yellow hairs, and on the ventral margin of labrum with a row of sparse yellow hairs. Mandible yellow but black distally. Maxillae and labial palpi yellow with short black hairs. Eyes divided by a transverse furrow. Antennae dark-brown, yellow basally. Club pyriform, dark-brown but with the basal 1/3 yellow.

**Thorax.** Black with long pale yellow hairs, the anterior and posterior margins of pronotum yellow, prescutum, scutum, scutellum of mesonotum and metascutellum black with two big yellow spots. The lateral part of thorax dark-brown with long dense white hairs.

**Wings.** Membrane hyaline. Forewing: Yellow basally, veins of C, M and 1/3 of the base of CuP and the inner margin to projection of anal area yellow, the rest of veins brown with rather short setae; axillary angle obtuse; pterostigma yellow with 3-4 cross veins; apical area beyond the vein Sc+R with 2-3 rows of cells, CuA area with 4-5 rows of cells. Hindwing: Shorter than forewing, the veins of C, M yellow, the CuP almost entirely yellow; pterostigma yellow with 3-4 cross veins, apical area beyond the vein Sc+R with 2-3 rows of cells, CuA area with 4-5 rows of cells.

**Legs.** Femur and tibia yellow but with the inner surface dark brown; the tarsi orange but the inner and distal portions dark brown. The claws and spurs red-brown. Hairs on femur long white. Inner surface of tibia with long black setae, but the outside with short black setae. Each segment of tarsi with short black spines distally.

**Abdomen.** Shorter than hindwing. Tergites black centrally, yellow-brown laterally, each tergite with two pairs of black transverse lines oriented from the center to the lateral margin. Sternites black centrally but yellow-brown laterally. The basis of abdomen with long dense white hairs, the rest with short black setae.

**Figure 12.**
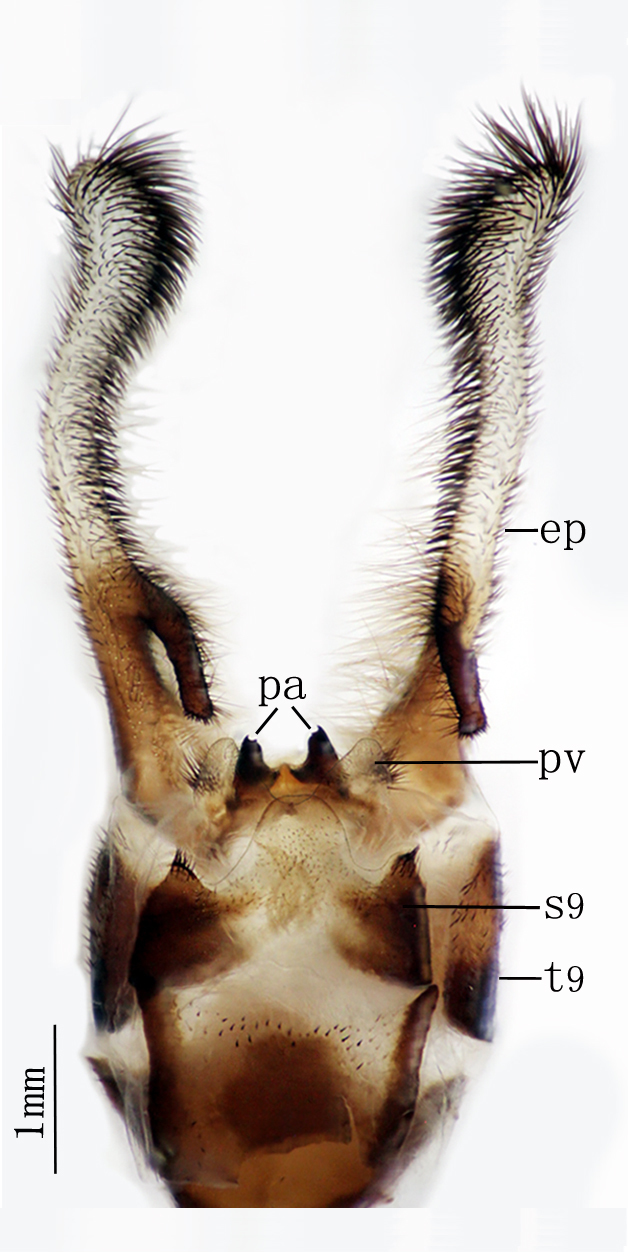
*Bubopsis tancrei* Weele, 1908, Abdomen terminal of male, ventral view.

**Figure 13.**
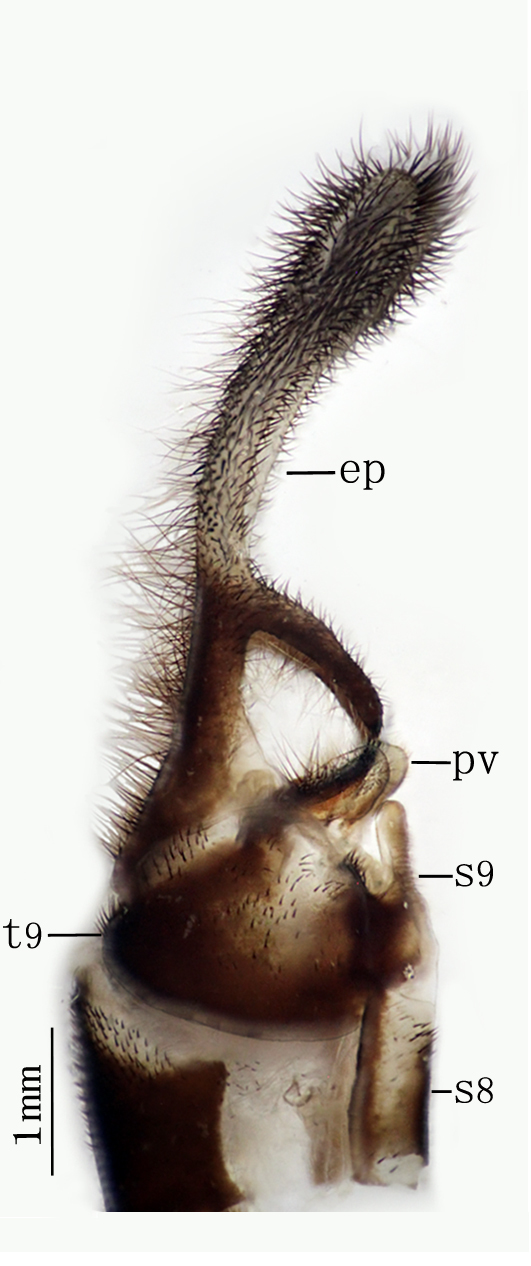
*Bubopsis tancrei* Weele, 1908, Abdomen terminal of male, lateral view.

**Male genitalia** Figs 12, 13 Ectoprocts elongate, almost as long as 6-9 abdominal segments together, with dense, dark-brown setae, and the basal 1/3 with a rod-shaped branch directed downward. Pulvini pale covered with several black gonosetae. Gonarcus connected with a pair of strongly sclerous parameres, the apex of parameres with two little teeth. Pelta absent.

**Female** Fig. 11

**Size.** Body length 26 mm. Antennae length 21 mm. Forewing length 32 mm. Hindwing length 28 mm. Abdomen length 16 mm.

**Figure 14.**
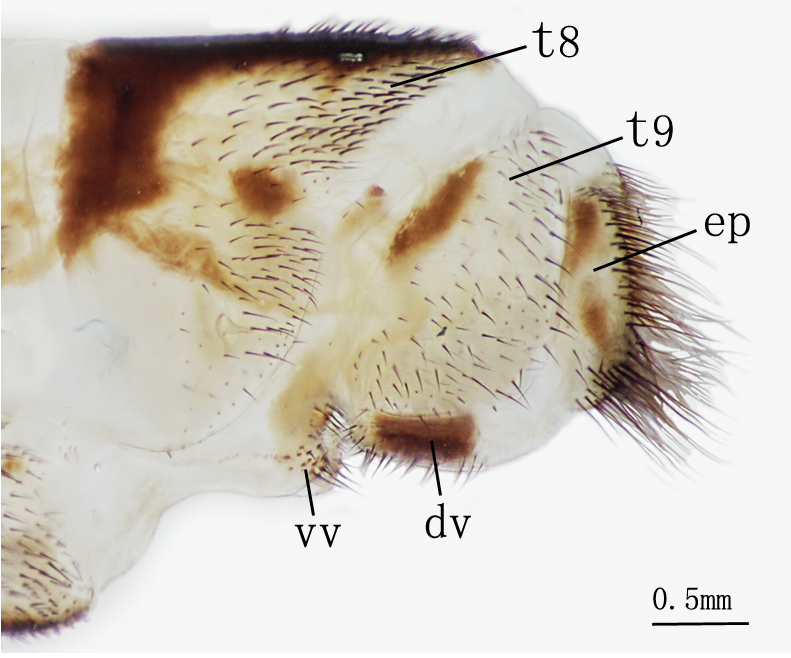
*Bubopsis tancrei* Weele, 1908, Abdomen terminal of female, lateral view.

**Figure 15.**
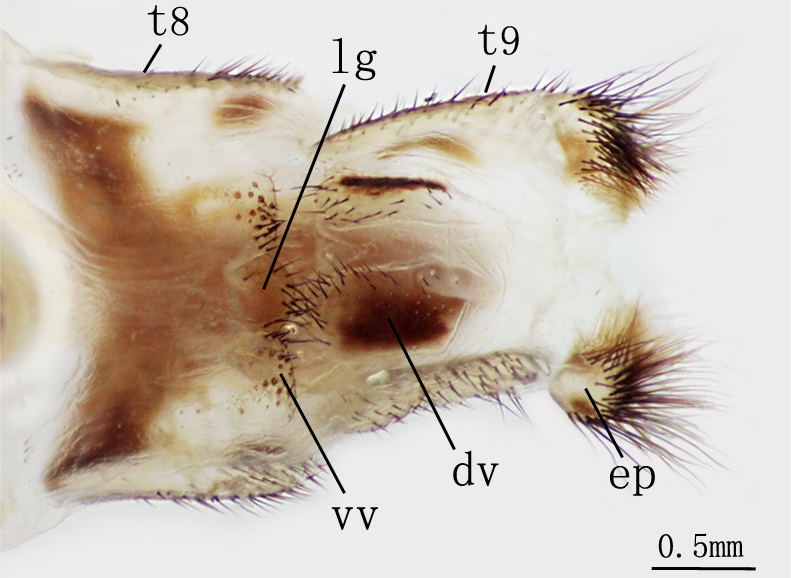
*Bubopsis tancrei* Weele, 1908, Abdomen terminal of female, ventral view.

**Female genitalia** Figs 14, 15 Ectoprocts elliptic in lateral view with long dense brown hairs. Distivalvae dark-brown with short brown setae. Linguella fused together, pale-brown with short brown setae. Ventrovalvae pale-brown with short brown setae. Interdens absent.

#### Distribution

China: Xinjiang; Kazakhstan, Turkmenistan, Uzbekistan.

#### Material examined

2♂，2♀, Urumchi, Xinjiang Province, 24-Ⅵ-1963, Huang Dawen, CAU-N200284, CAU-N200285, CAU-N200274, CAU-N200283.

#### Distribution map

Fig. 16

## Supplementary Material

XML Treatment for Nousera

XML Treatment for Nousera gibba

XML Treatment for Bubopsis

XML Treatment for Bubopsis tancrei

## Figures and Tables

**Figure 1.**
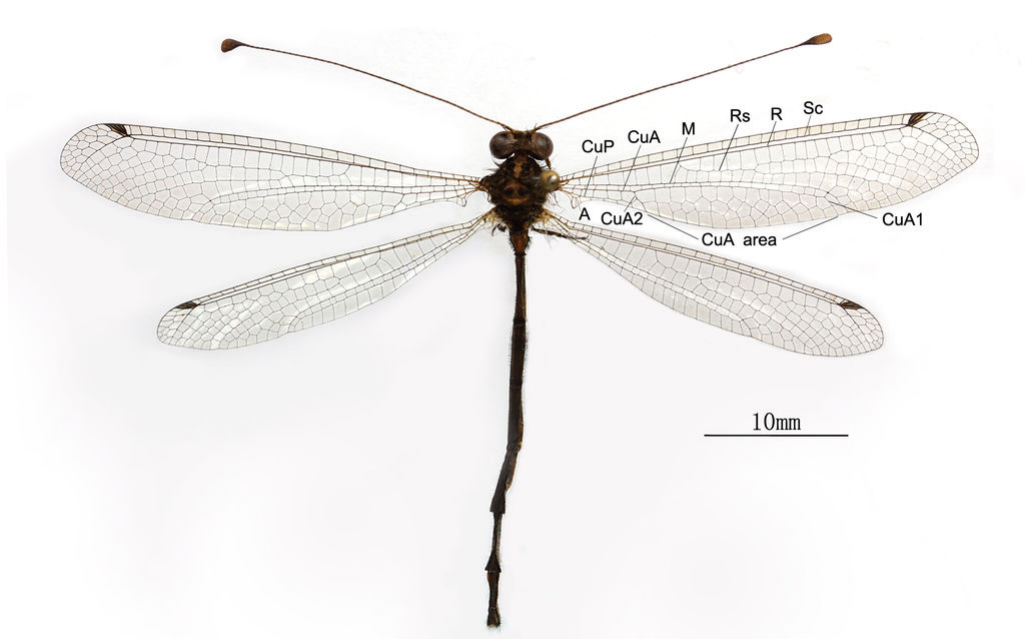
*Nousera gibba* Navás, 1923, Male habitus.

**Figure 2.**
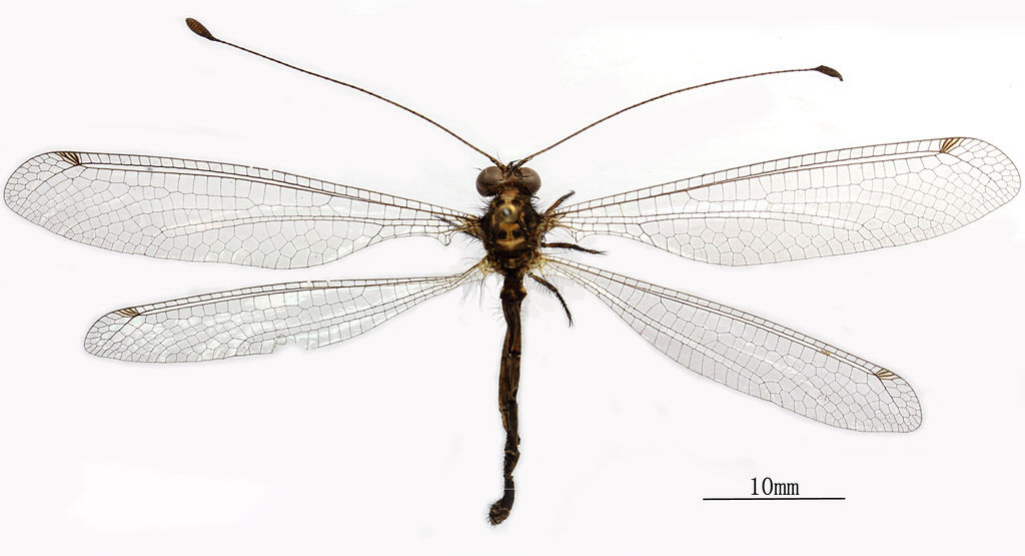
*Nousera gibba* Navás, 1923, Female Habitus.

**Figure 10.**
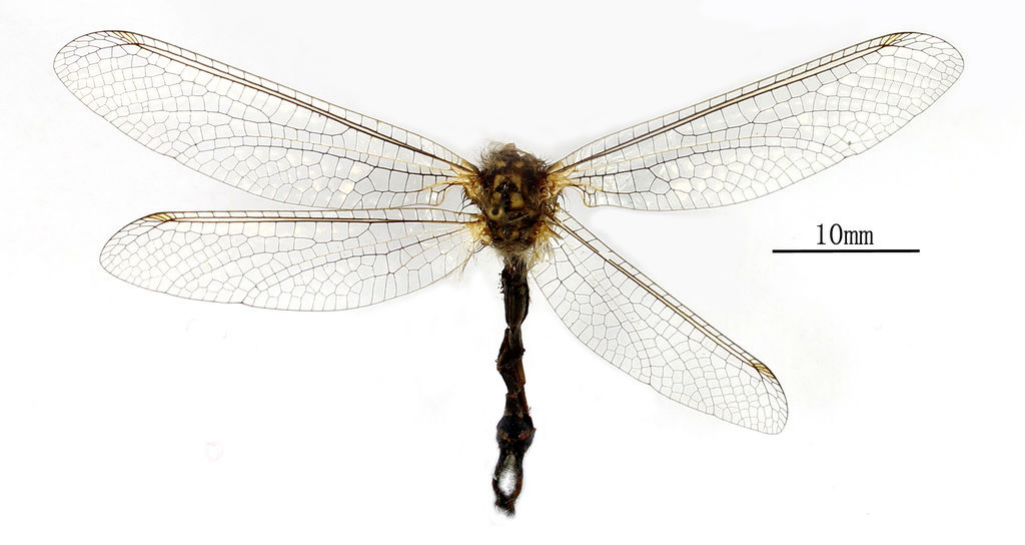
*Bubopsis tancrei* Weele, 1908, Male Habitus.

**Figure 11.**
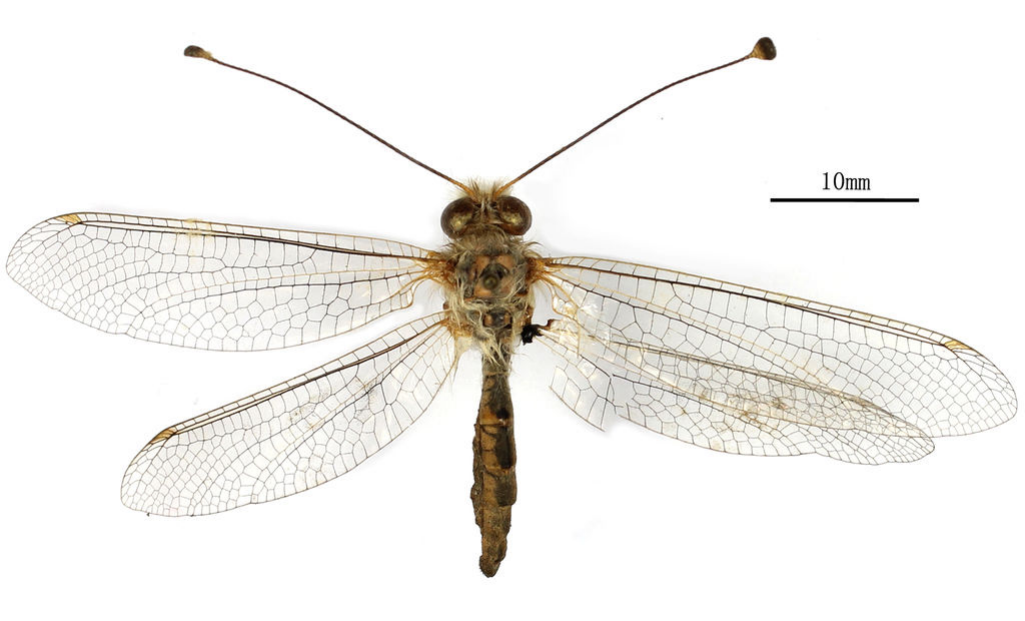
*Bubopsis tancrei* Weele, 1908, Female Habitus.

**Figure 16.**
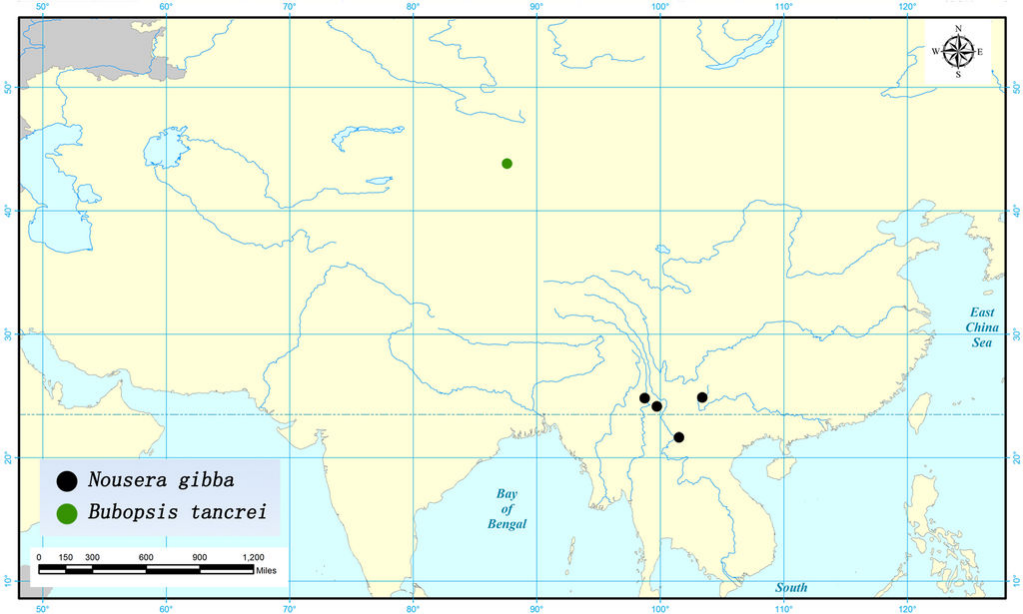
Distribution of *Nousera gibba* and *Bubopsis tancrei* in China.
